# Death receptor 5 promotes tumor progression in gastric cancer

**DOI:** 10.1002/2211-5463.13725

**Published:** 2023-11-14

**Authors:** Junbing Chen, Lin Li, Longtao Huangfu, Hong Du, Xin Ji, Xiaofang Xing, Jiafu Ji

**Affiliations:** ^1^ Department of Gastrointestinal Cancer Translational Research, Key Laboratory of Carcinogenesis and Translational Research (Ministry of Education/Beijing) Peking University Cancer Hospital & Institute Beijing China; ^2^ Department of Gastroenterology, Aerospace Center Hospital Peking University Aerospace School of Clinical Medicine Beijing China; ^3^ Gastrointestinal Cancer Center, Key Laboratory of Carcinogenesis and Translational Research (Ministry of Education/Beijing) Peking University Cancer Hospital and Institute Beijing China

**Keywords:** apoptosis, death receptor 5, gastric cancer, nuclear localization, TRAIL receptors

## Abstract

Death receptor 5 (DR5) can inhibit malignant proliferation via tumor necrosis factor‐related apoptosis‐inducing ligand (TRAIL)‐induced apoptosis in many cancers. Here we examined the expression and sublocalization of DR5 in gastric cancer, as well as its effects on clinical prognosis and cellular processes. Our analysis included a cohort of 240 gastric cancer patients. Bioinformatic analysis showed a significant correlation between DR5 and DNA replication, tumor mutation burden (TMB), and tumor stemness. Unlike death receptor 4 (DR4TRAIL‐R1), DR5 was expressed in the cytoplasm and nucleus, and was found to be positively correlated with lymphovascular invasion, lymph node metastasis, and TNM stage. Patients with positive DR5 had worse overall survival (OS) (*P* = 0.006). The multivariate Cox model showed that DR5 is an independent poor prognostic factor (hazard ratio = 1.693). Furthermore, knockdown of DR5 inhibited aggressive behaviors, including proliferation and metastasis in gastric cancer cells, and inhibited lung metastasis *in vivo*. In summary, nuclear localization of DR5 expression is a poor prognosis factor in gastric cancer and promotes growth, invasion, and metastasis of tumor cells *in vitro* and *in vivo*.

## Graphical abstract

DR4 and DR5 are generally expressed on the cell membrane and mediate TRAIL‐induced apoptosis. However, in gastric cancer cells nuclear translocation of DR5 is often observed. The absence of DR5 on the cell membrane fails to mediate TRAIL‐induced apoptosis, and nuclear translocation promotes the progression of gastric cancer through relevant mechanisms, thereby affecting prognosis.

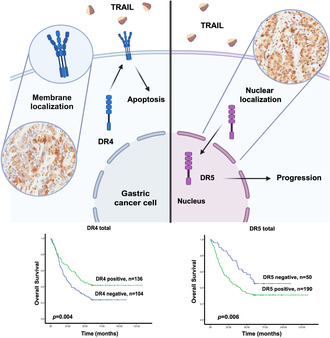

**Abbreviations**
BSA: bovine serum albumin
CCLE: Cancer Cell Line Encyclopedia
DD: death domains
DEG: differentially expressed gene
DISC: death‐inducing signaling complex
DR4, TNFRSF10A, TRAIL‐R1: death receptor 4
DR5, TNFRSF10B, TRAIL‐R2: death receptor 5
FBS: fetal bovine serum
FFPE: formalin‐fixed paraffin‐embedding
FLIP: FLICE inhibitory protein
GO: Gene Ontology
ICI: immune checkpoint inhibitor
IHC: immunohistochemistry staining
KEGG: Kyoto Encyclopedia of Genes and Genomes
LN: lymph node
MSI: microsatellite instability
NLS: nuclear localization signals
NSCLC: non‐small‐cell lung cancer
NT: adjacent normal tissue
OCLR: one‐class logistic regression
OS: overall survival
PDAC: pancreatic ductal adenocarcinoma
PT: primary tumor
PTX: paclitaxel
rhTRAIL: recombinant human TRAIL
SD: standard deviation
SDS/PAGE: SDS polyacrylamide gel electrophoresis
TBST: tris‐buffered saline tween‐20
TCGA: The Cancer Genome Atlas
TMA: tissue microassay
TMB: tumor mutation burden
TNF: tumor necrosis factor
TNM: tumor‐node‐metastasis
TRAIL: tumor necrosis factor‐related apoptosis‐inducing ligand

Gastric cancer is a leading cause of cancer‐associated deaths due to its malignancy and heterogeneity. Although multimodal therapies such as chemotherapy, surgery, targeted therapy, and immunotherapy improve survival outcomes of gastric cancer patients, there are many patients suffering from local recurrence and distant metastasis [1]. Many factors play a positive or negative role in tumorigenesis and metastasis in gastric cancer, and one of the important factors is tumor necrosis factor (TNF)‐related apoptosis‐inducing ligand (TRAIL), which is a kind of type II transmembrane protein of TNF superfamily members [2].

Different from other TNF members, TRAIL has five known receptors identified in humans, including death receptor 4 (DR4, TNFRSF10A, TRAIL‐R1), death receptor 5 (DR5, TNFRSF10B, TRAIL‐R2), DcR1 (TRAIL‐R3), DcR2 (TRAIL‐R4), and osteoprotegerin (TRAIL‐R5). Death receptor 4 and DR5 have been confirmed to induce apoptosis of tumor cells through their cytoplasmic death domains (DD), and finally inhibit malignant proliferation in many cancers [3, 4]. DcR1 and DcR2 are considered decoy receptors that can attenuate DR4‐ and DR5‐induced effects of apoptosis because of a lack of DD [5, 6]. Due to DR4 and DR5 apoptotic effects, many targeted therapies and combined therapies had been developed. In our previous study, we found paclitaxel (PTX) could enhance TRAIL‐induced apoptosis by upregulation of death receptors and downregulation of the antiapoptotic protein [7]. Moreover, we also reported that Trichostatin A could strengthen the TRAIL‐induced apoptotic effect via inhibition of ERK/FOXM1 [8].

However, this was inconsistent with the biological function of promoting apoptosis that high expression of DR5 was correlated with better or worse survival outcomes in some cancers, such as lung cancer (NSCLC) [9, 10, 11]. Therefore, there are many unknown mechanisms of DR5 in different cancers. It was reported that DR5 was translocated into the nucleus by importin β1 [12], and DR5 in the nucleus could inhibit the maturation of let‐7 and promote the proliferation of pancreatic ductal adenocarcinoma (PDAC) cells [13].

In our previous study, we reported that susceptibility of gastric cancer cells to TRAIL was mainly ascribed to surface expression of DR4 in the cytomembrane rather than DR5 [7]. In melanoma, it was reported that DR5 could activate the NF‐κB pathway to induce metastasis instead of an apoptosis effect [14]. In non‐small‐cell lung cancer and PDAC, DR5, rather than DR4, was reported to be correlated with invasion and metastasis by mediating a KRAS‐induced effect [15]. Likewise in breast cancer, DR5 was reported to promote metastasis [16]. Therefore, in gastric cancer we postulated that high membranous expression of DR4 can predict a better prognosis because it can mediate TRAIL‐induced cytotoxicity, but high DR5 expression in gastric cancer cells may induce malignant effects by a noncanonical pathway due to its partial sublocalization in the nucleus. In this study, to reveal the different roles of DR4 and DR5 in gastric cancer, we aimed to investigate the expression, sublocalization, and the clinical prognosis of DR4 and DR5 in gastric cancer tissue, and further confirm the high nucleus expression of DR5 in gastric cancer cells can promote tumor cells migration, invasion, and distant metastasis *in vitro* and *in vivo*.

## Results

### DR expression was associated with immune resistance in gastric cancer by bioinformatic analysis

**Fig. 1:**
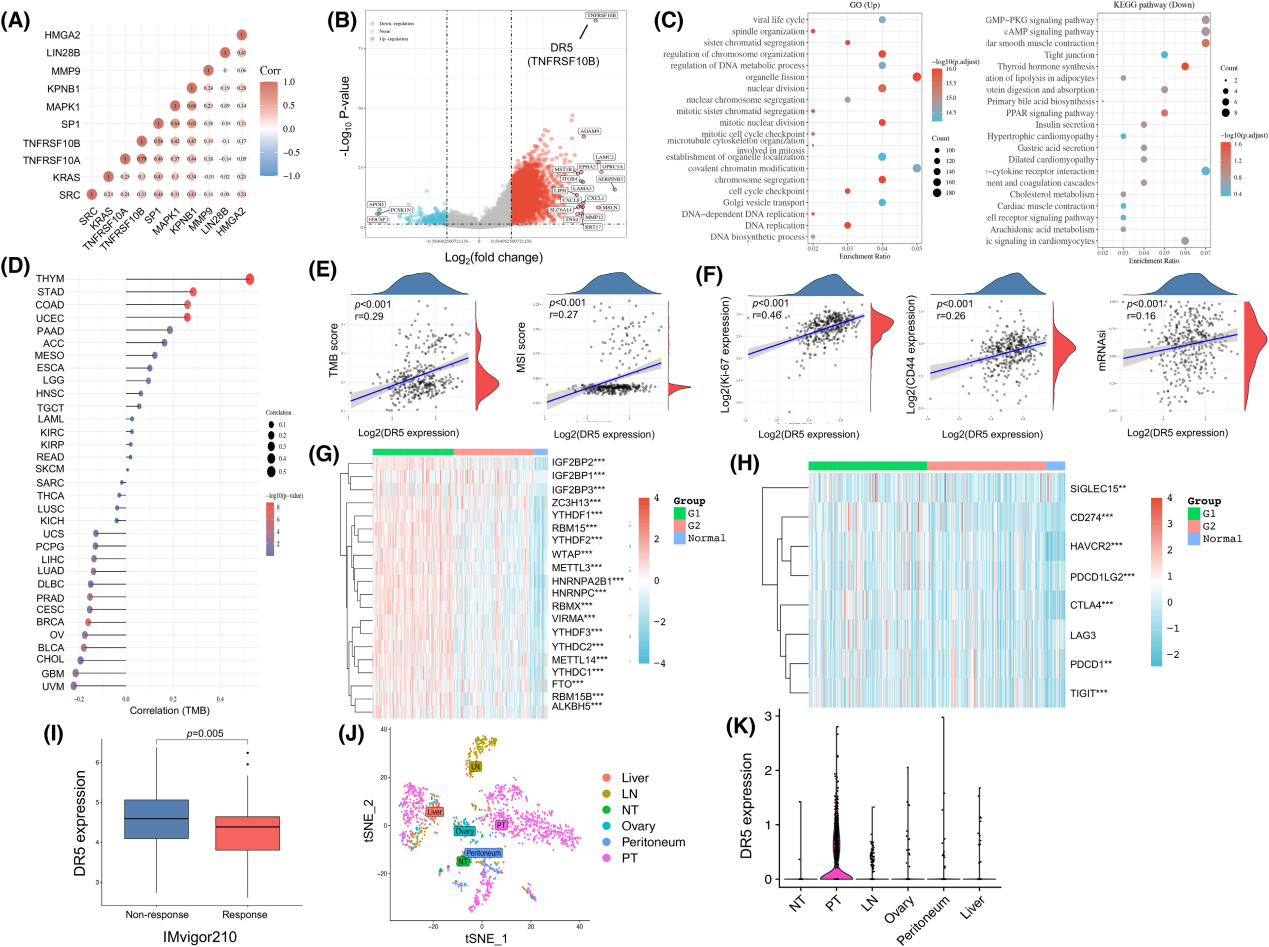
Bioinformatic analysis of death receptor 5 (DR5) (TNFRSF10B) expression in gastric cancer. (A) Association matrix of genes mRNA expression between DR5 and malignant genes in the Cancer Genome Atlas (TCGA) dataset. (B) The volcano plot of differentiated expressed genes as the 25% top highest versus the 25% lowest expression of DR5. (C) Gene Ontology (GO) annotation of differentiated expressed genes in upregulation passage and Kyoto Encyclopedia of Genes and Genomes (KEGG) annotation of differentiated genes in downregulation passage. (D) The correlation of DR5 and tumor mutation burden (TMB) in pan‐cancer comparison of TCGA. (E) The correlation plots between DR5 and TMB score, and microsatellite instability (MSI) score. (F) The correlation plots between DR5 and MKI67, CD44, and mRNAsi (stemness index) score. (G) The heatmap of m6A‐related genes in patients' group with high (G1) or low (G2) expression of DR5, and normal group. (H) The heatmap of immune checkpoint in patients' group with high (G1) or low (G2) expression of DR5, and normal group. (I) The comparison of DR5 expression between groups of nonresponse and response in cancer patients treated with the immune checkpoint inhibitor (IMvigor210). (J) The single‐cell RNA‐Seq (scRNA‐Seq) tSNE reduction in the subcluster of epithelial or tumor cells (GSE163558). (K) The scRNA‐Seq‐based comparison of DR5 expression in normal cells and primary or metastatic gastric cancer cells (LN, lymph node; NT, adjacent normal tissue). Spearman correlation analysis, Mann–Whitney *U*‐test, and Kruskal–Wallis test were used. **P‐value < 0.01 and ***P‐value < 0.001.

The DR5 mRNA expression in STAD of the TCGA dataset (*n* = 375) is analyzed in Fig. 1. DR5 was coexpressed, with many reported malignant or nuclear localization‐related genes including SRC, KPNB1, SP1, MAPK1, KRAS, MMP9, HMGA2, and LIN28B (Fig. 1A). Moreover, DR5 had a higher correlation score than DR4 among these genes. We divided the STAD patients into two groups, with the top 25% highest (*n* = 94) and 25% lowest (*n* = 94) DR5 expression, respectively. The differentially expressed genes (DEGs) are shown in the volcano plot (Fig. 1B). Gene Ontology (GO) and KEGG annotation passages of DEG showed that DR5‐related up‐DEGs were involved in DNA replication, regulation of chromosome organization, chromosome segregation, and nuclear division in GO annotation, which revealed that DR5 was associated with the process of nuclear localization, DNA regulation, mitosis, and segregation. In addition, DR5‐related down‐DEGs were involved in tight junctions, which revealed that DR5 was related to cell invasion and migration (Fig. 1C). DR5 mRNA expression in STAD had the top correlation with TMB in the pan‐cancer comparison of the TCGA dataset (Fig. 1D). DR5 expression was related to TMB (correlation = 0.29, 95% confidence interval [CI] = 0.19–0.38, *P* < 0.001) and MSI (correlation = 0.27, 95% CI = 0.17–0.36, *P* < 0.001) in STAD (Fig. 1E). Moreover, DR5 expression was also related to the expression of MKI67 and CD44, and stemness index (mRNAsi), which revealed the correlation between DR5 and tumor stemness (Fig. 1F). We analyzed the correlation between DR5‐ and m6A‐related genes and showed that patients with high DR5 expression had a significantly higher expression of genes involving writers, erasers, and readers in m6A pathways (Fig. 1G). Besides, we also analyzed the correlation between DR5 and the immune checkpoint and showed that patients with high DR5 expression had a significantly higher expression of SIGLEC15, CD274 (PD‐L1), HAVCR2 (TIM3), PDCD1LG2 (PD‐L2), CTLA4, PDCD1 (PD‐1), and TIGIT than patients with low DR5 expression, and normal persons, which means that high DR5 expressed in patients might have greater immune resistance (Fig. 1H). Patients who respond to ICI had significantly lower expression of DR5 in urothelial cancer with anti‐PD‐L1 therapy (Log_2_ Fold‐Change = −0.28, *P* = 0.005) (Fig. 1I) [17]. However, DR4 expression was not correlated with the response to ICI (*P* = 0.063) (Fig. S1). The scRNA‐Seq analysis showed that DR5 was significantly highly expressed in primary tumor cells and partially expressed in distant metastatic tumor tissues, including lymph node, ovary, peritoneum, and liver. In addition, DR5 was barely expressed in adjacent normal tissue (Fig. 1J,K).

### Relationship between DR4/DR5 expression and clinicopathological features in gastric cancer patients

**Fig. 2:**
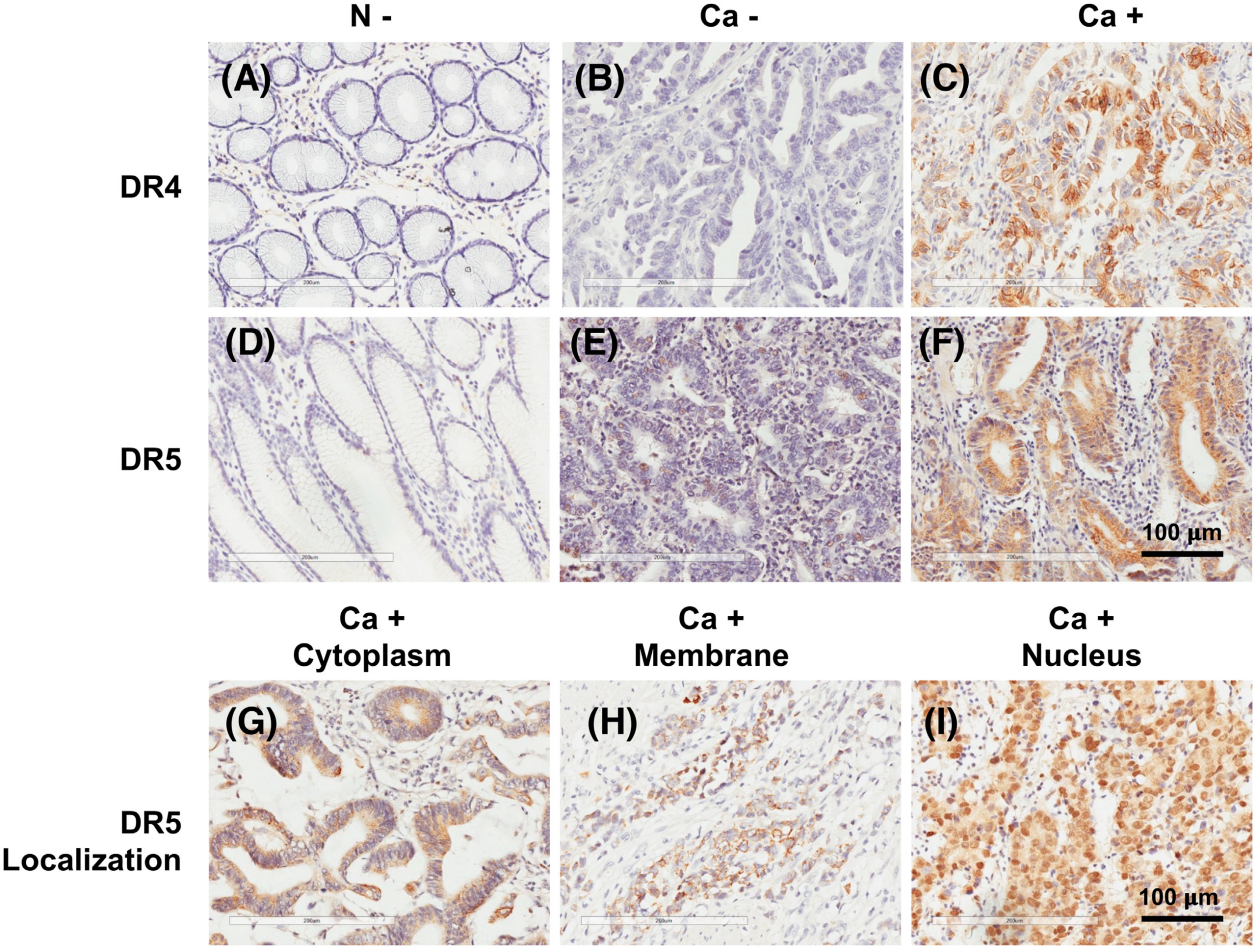
Immunohistochemical staining of death receptor 4 (DR4) and DR5 expression in gastric cancer. (A–C) DR4 expression in normal (A) and tumor tissue from gastric cancer patients (B,C). (D–F) DR5 expression in normal (D) and tumor tissue from gastric cancer patients (E,F). (G–I) DR5 sublocalization in cytoplasm (G), membrane (H), and nucleus (I) in gastric tissue. Scale bars, 100 μm (A–I).

The immunohistochemical staining of specimens from 240 gastric cancer patients showed that DR4 was expressed in the membrane, but DR5 in multiple locations in gastric cancer tissue (Fig. 2A–I). Figure 2C shows DR4 sublocalization in the membrane, and Fig. 2G–I shows DR5 sublocalization in the cytoplasm, membrane, and nucleus, respectively, in gastric cancer tissue. Statistical analysis indicated that DR4 expression was negatively related to the depth of invasion and distant metastasis, and DR5 expression was positively related to lymphovascular invasion and lymph node metastasis (Table 1). Female patients showed a lower DR4 expression rate (39.4% neg vs. 22.1% pos, *P* = 0.003). Patients with poor differentiation showed a lower DR4 expression rate (75.0% of neg vs. 61% of pos, *P* = 0.022). Patients with other histological types except adenocarcinoma showed a lower DR4 expression rate (26.9% of neg vs. 10.3% of pos, *P* = 0.001). Patients with the depth of invasion at T3 and T4 showed lower DR4 expression rate (91.3% of neg vs. 81.6% of pos, *P* = 0.032). Patients with distant metastasis at M1 showed a lower DR4 expression rate (17.3% of neg vs. 8.1% of pos, *P* = 0.030). However, patients with lymphovascular invasion showed a higher DR5 expression rate (46.0% of neg vs. 62.6% of pos, *P* = 0.033). Patients with lymph node metastasis showed a higher DR5 expression rate than the absence of metastasis (64.0% of neg vs. 81.6% of pos, *P* = 0.008). The result showed that patients with III–IV of TNM stage compared with I‐II of stage had a higher DR5 expression rate (55.3% of neg vs. 77.7% of pos, *P* = 0.002), but the DR4 expression rate was similar (76.0% of neg vs. 72.1% of pos, *P* = 0.496).

**Table 1:** Relationship between death receptor 4 (DR4)/DR5 expression and clinicopathological features of patients with gastric cancer. The data in parentheses are the percentage of negative or positive gene expression in different subgroups of features. The data outside the parentheses were patients' number used for statistical comparison. The chi‐squared test was used to compare the expression in different clinicopathological features. *P* < 0.05 was statistically significant and is labeled with an asterisk.

Clinicopathological features	DR4 expression		*P*‐value	DR5 expression		*P‐*value
	Negative (%)	Positive (%)		Negative (%)	Positive (%)	
Gender						
Male	63 (60.6)	106 (77.9)	0.003*	34 (68.0)	135 (71.1)	0.674
Female	41 (39.4)	30 (22.1)		16 (32.0)	55 (28.9)	
Age, years						
≤ 60	57 (54.8)	63 (46.3)	0.193	27 (54.0)	93 (48.9)	0.525
> 60	47 (45.2)	73 (53.7)		23 (46.0)	97 (51.1)	
Differentiation						
Well and moderate	26 (25.0)	53 (39.0)	0.022*	12 (24.0)	67 (35.3)	0.132
Poor	78 (75.0)	83 (61.0)		38 (76.0)	123 (64.7)	
Histologic type						
Adenocarcinoma	76 (73.1)	122 (89.7)	0.001*	40 (80.0)	158 (83.2)	0.601
Others	28 (26.9)	14 (10.3)		10 (20.0)	32 (16.8)	
Lymphovascular invasion						
Absent	41 (39.4)	57 (41.9)	0.698	27 (54.0)	71 (37.4)	0.033*
Present	63 (60.6)	79 (58.1)		23 (46.0)	119 (62.6)	
Depth of invasion						
T1 + T2	9 (8.7)	25 (18.4)	0.032*	8 (16.0)	26 (13.7)	0.676
T3 + T4	95 (91.3)	111 (81.6)		42 (84.0)	164 (86.3)	
Lymph node metastasis						
No	24 (23.1)	29 (21.3)	0.746	18 (36.0)	35 (18.4)	0.008*
Yes	80 (76.9)	107 (78.7)		32 (64.0)	155 (81.6)	
Distant metastasis						
M0	86 (82.7)	125 (91.9)	0.030*	44 (88.0)	167 (87.9)	0.984
M1	18 (17.3)	11 (8.1)		6 (12.0)	23 (12.1)	
TNM stage						
I–II	25 (24.0)	38 (27.9)	0.496	21 (44.7)	42 (22.3)	0.002*
III–IV	79 (76.0)	98 (72.1)		26 (55.3)	146 (77.7)	

### DR5 expression predicted worse survival in gastric cancer patients

**Fig. 3:**
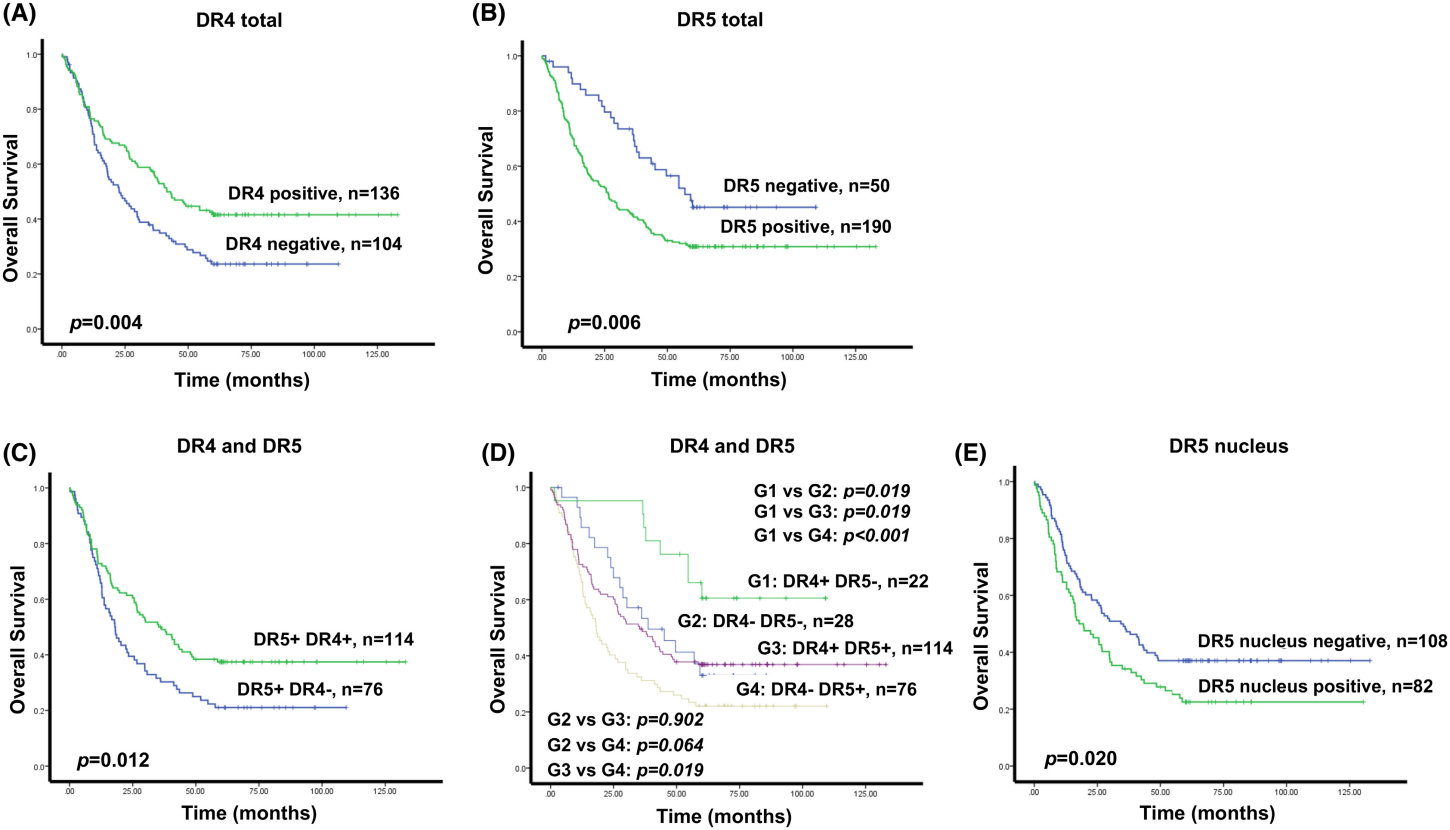
Kaplan–Meier curves estimated the influence of death receptor 4 (DR4)/DR5 expression in 240 gastric cancer patients' tissues on overall survival (OS). (A) OS curves stratified by DR4 expression. (B) OS curves stratified by DR5 expression. (C) OS curves stratified by DR4 expression in a subpopulation with DR5‐positive expression. (D) OS curves stratified by combined expression of DR4 and DR5. The log‐rank test and Benjamini–Hochberg methods were performed for pairwise comparison in multiple survival curves. (E) OS curves stratified by DR5 nucleus expression.

To reveal the prognosis of DR4 and DR5 expression in gastric cancer, Kaplan–Meier curves indicated that patients with DR4‐positive expression had better overall survival (OS) outcomes (*n* = 136, 56.7%, *P* = 0.004) (Fig. 3A), but DR5‐positive expression had worse OS outcomes (*n* = 190, 79.2%, *P* = 0.006) (Fig. 3B). However, in the subgroup of patients with DR5‐positive expression, DR4‐positive expression still had a better survival outcome (*n* = 114, 60.0%, *P* = 0.012) (Fig. 3C). In addition, patients with DR4‐positive expression and DR5‐negative expression (*n* = 22, 9.2%) had relatively better survival outcomes, and patients with DR4‐negative expression and DR5‐positive expression (*n* = 76, 31.7%) had relatively worse survival outcomes. Based on the log‐rank test and Benjamini–Hochberg method for the *post hoc* test, compared with the G1 group (patients with DR4‐positive and DR5‐negative expression), G2 group patients (DR4 and DR5 negative expression) and G3 group patients (DR4 and DR5 positive expression) both had a worse survival outcome (*P* = 0.019), but G4 group patients (DR4‐negative and DR5‐positive expression) had a significantly worse survival outcome (*P* < 0.001). Compared with the G2 group, the G3 group had no statistical difference (*P* = 0.092), but the G4 group had an approaching significance (*P* = 0.064). Compared with the G3 group, the G4 group had a significantly worse survival outcome (*P* = 0.019) (Fig. 3D). As TRAIL receptor‐inducing apoptosis, surface DR4 and DR5 expression in the cytomembrane usually promoted tumor cell death and is beneficial for patients' survival. Although DR4 and DR5 expression were observed in the cytomembrane, DR5 was also expressed in the nucleus in gastric cancer tissues, which might promote tumor cells' malignancy. Therefore, we further analyzed the expression of DR5 in the nucleus in the subgroup of DR5‐positive patients. Patients with positive DR5 nucleus expression had worse survival outcomes than negative expression (*n* = 82, 43.16% vs. *n* = 108, 56.84%; *P* = 0.020) (Fig. 3E).

The results of the univariate and multivariate Cox's models for the OS of gastric cancer patients are shown in Table 2. The significant variables in the Cox model analysis included DR4, DR5, lymphovascular invasion, depth of invasion, lymph node metastasis, distant metastasis, and age. The risk of patients with positive DR4 expression was 0.589 (95% CI, 0424–0.817) lower than those with negative DR4 expression (*P* = 0.002). However, the risk of patients with positive DR5 expression was 1.693 times (95% CI, 1.099–2.606) higher than those with negative DR5 expression (*P* = 0.017). Other variables such as lymphovascular invasion, deeper invasion, lymph node metastasis, and age older than 60 had a significantly higher risk, as expected.

**Table 2:** Univariate and multivariate Cox's models for overall survival of GC patients. *P* < 0.05 was statistically significant and is labeled with an asterisk.

Variables	Univariate analysis			Multivariate analysis		
	Hazard ratio	95% CI	*P*‐value	Hazard ratio	95% CI	*P*‐value
DR4						
Positive vs. Negative	0.631	0.461–0.864	0.004*	0.589	0.424–0.817	0.002*
DR5						
Positive vs. Negative	1.792	1.176–2.732	0.007*	1.693	1.099–2.606	0.017*
Lymphovascular invasion						
Present vs. absent	2.008	1.428–2.779	0.000*	1.482	1.034–2.124	0.035*
Depth of invasion						
T_3_ + T_4_ vs. T_1_ + T_2_	3.501	1.882–6.435	0.000*	2.093	1.105–3.966	0.025*
Lymph node metastasis						
Yes vs. no	3.378	2.049–5.493	0.000*	2.220	1.305–3.777	0.003*
Distant metastasis						
M1 vs. M0	2.859	1.889–4.340	0.000*	2.310	1.511–3.531	0.000*
Gender						
Female vs. male	0.964	0.685–1.368	0.852	0.822	0.577–1.170	0.276
Age						
> 60 vs. ≤ 60	1.537	1.128–2.117	0.007*	1.551	1.119–2.110	0.008*

### Interference to DR5 expression impacted the gastric cancer cells' malignancy

**Fig. 4:**
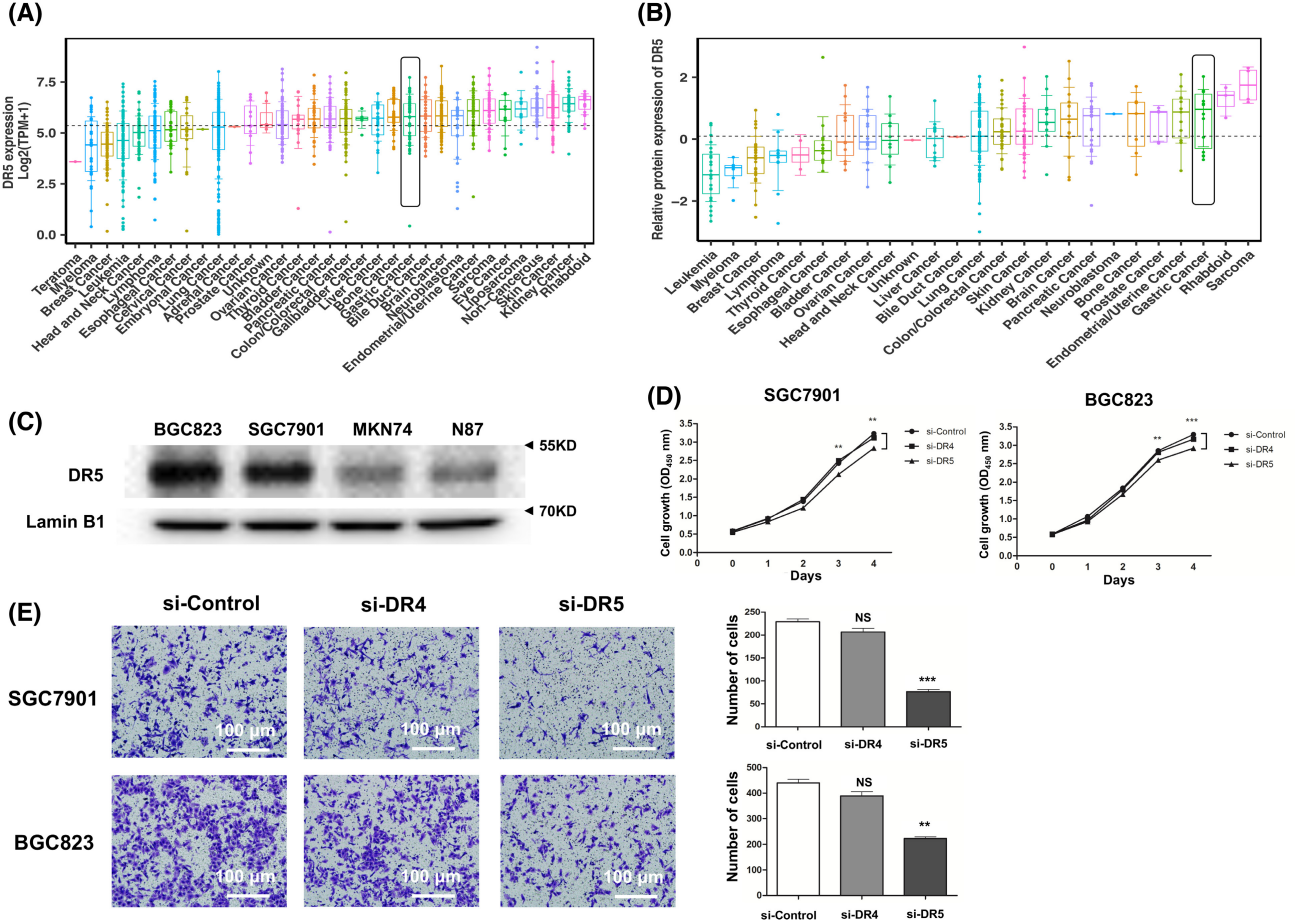
Interference in death receptor 5 (DR5) expression impacted the gastric cancer cells malignance. (A,B) DR5 (TNFRSF10B) transcriptomic and proteomic expression in cell lines. (C) Extraction of nucleus proteins for detection of endogenous DR5 expression by western blot, and nuclear membrane structural components ‘Lamin B1’ as a nucleus biomarker. (D,E) Cell viability assay using CCK‐8 and cell migration assay using transwell chamber in SGC7901 and BGC823 transfected with siRNA or negative control (si‐Control). The independent experiments were performed (*n* = 3). The Mann–Whitney *U*‐test was used. **P‐value < 0.01 and ***P‐value < 0.001. Error bars represent the standard deviation. Scale bars, 100 μm (E).

In the bioinformatics analysis of cell lines expression of DR5 from TCGA and CCLE datasets, DR5 had a relatively high expression among cancers (Fig. 4A) and in most gastric cancer cells (Fig. 4B). To further confirm the biological functions of nucleus DR5 expression in gastric cancer, we constructed cells with RNA interference to DR5 to reveal its influence on cells' malignancy. BGC823 and SGC7901 were selected because of their high extracted protein expression of DR5 in the nucleus using western blot (Fig. 4C, Fig. S2). Cell viability assays using CCK‐8 showed that the cell growth ability in both SGC7901 and BGC823 with siRNA to DR5 was decreased (Fig. 4D). Likewise, in the transwell chamber assay of migration, siRNA to DR5 rather than DR4 in both SGC7901 and BGC823 impaired the cells' ability to migrate (Fig. 4E).

### Knockdown of DR5 inhibits lung metastasis of gastric cancer cells in an *in vivo* xenograft mouse model

**Fig. 5:**
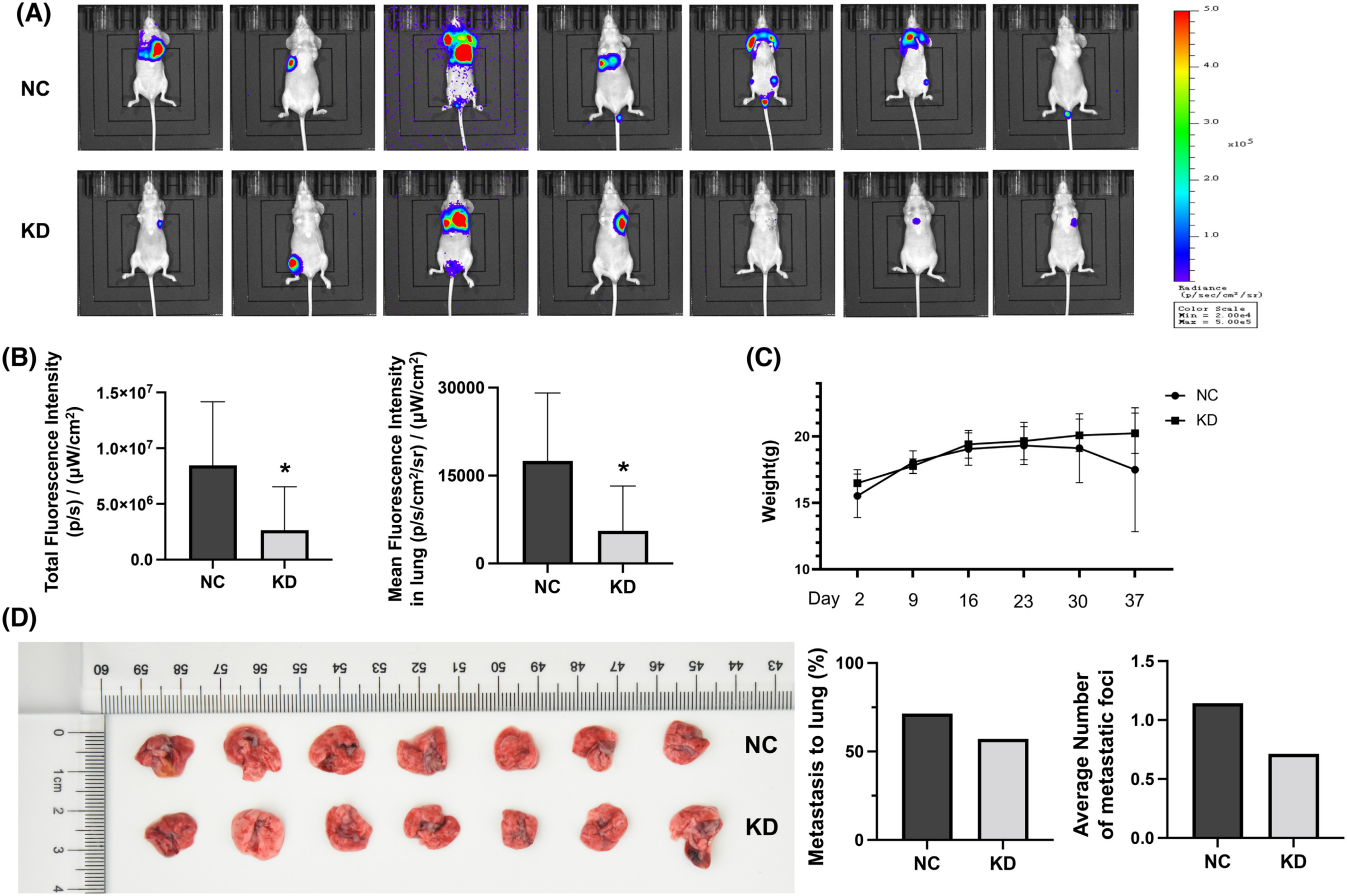
Knockdown of death receptor 5 (DR5) inhibited lung metastasis of gastric cancer cells *in vivo* xenograft mouse model (*n* = 7 each). (A) Fluorescence imaging *in vivo* of mice injected with NC (negative control) or KD (knockdown)‐DR5 BGC823 cells. (B) The comparison of fluorescence intensity in total (*P* = 0.046) and lung (*P* = 0.042) area of mice with potential metastasis between NC and KD of DR5. (C) The weight curves of mice injected with NC or KD‐DR5 cells (*P* = 0.165). (D) The lung tissues dissected from injected mice, the percentage of metastasis to lung, and the average number of metastatic foci in mice (*P* = 0.403). The Student's *t*‐test was performed. Error bars represent the standard deviation. *P* < 0.05 was statistically significant and is labeled with an asterisk.

*In vivo* xenograft mice injected with gastric cancer cells at the tail showed knockdown of DR5 attenuated BGC823 cells' metastasis to the lungs (Fig. 5A,B). The knockdown group showed significantly lower fluorescence intensity in total body area (*P* = 0.046) and lung area (*P* = 0.042). As shown in Fig. 5C, the weights of mice in the NC group were decreased indistinctly compared with that in the KD group (*P* = 0.165). The tissue specimens and radiant efficiency showed that there were fewer metastatic cells of BGC823 in the lung with knockdown of DR5 (Fig. 5D). The percentage of metastasis to lung in the negative control and knockdown group was, respectively, 71.40% and 57.10%. The average number of metastasis foci of body in the negative control group was 1.143, and in the knockdown group was 0.714.

## Discussion

In this study we found that the expression of DR5 (TRAILR2, TNFRSF10B) presented a malignant phenotype as an independent risk factor for poor prognosis in gastric cancer patients, which was different from DR4 (TRAILR1, TNFRSF10A). Bioinformatic analysis of DR5 in a gastric cancer cohort (TCGA‐STAD) showed DR5‐related up‐DEGs were involved in DNA replication and nuclear division, and down‐DEGs were involved in tight junction, which indicated DR5 in gastric cancer cells might play a role in the pathways of proliferation and metastasis. In addition, DR5 was also correlated with TMB, MSI, expression of Ki67 and CD44, and tumor stemness in gastric cancer, which need further deep research. In the study of the correlation of gene expression, we found gastric cancer patients with a relatively high expression of DR5 also had a higher expression of m6A‐related genes and immune checkpoint genes, which indicated that DR5 might be involved in the post‐transcriptional modification and immune resistance. The patient resistance to ICI therapy presented a higher expression of DR5, which indicated the potential role of DR5 in immune resistance. scRNA‐Seq results showed that DR5 in tumor cells was higher than those in normal epithelial cells, which indicated that high DR5 expression existed in both primary and metastatic tumor cells. In our cohort of 240 gastric cancer patients, DR5 expression showed a significantly positive correlation with vascular invasion (*P* = 0.033), lymph node metastasis (*P* = 0.008), and TNM staging (*P* = 0.023), which indicated that DR5 might be involved in invasion and metastasis in gastric cancer. However, contrary to DR5, high DR4 expression in gastric cancer patients showed a significantly lower depth of invasion (*P* = 0.032) and distant metastasis (*P* = 0.030), which was consistent with its biological function involving mediating apoptotic signaling pathways. The univariate and multivariate Cox model analysis showed that high DR4 expression was a protective prognosis factor (hazard ratio [HR] = 0.589, 95% CI = 0.424–0.817, *P* = 0.002), but DR5 expression was an independent risk factor (HR = 1.693, 95% CI = 1.099–2.606, *P* = 0.017). The survival analysis showed that DR4‐positive patients had a better OS (*P* = 0.004), but DR5‐positive patients had a worse OS (*P* = 0.006). Moreover, unlike DR4, we also found the special localization of DR5 in the cell nucleus of gastric cancer specimens. The immunohistochemistry (IHC) of DR5 in the nucleus in 190 gastric cancer patients showed that patients with positive DR5 expression in the nucleus of gastric cancer cells had a worse OS outcome (*P* = 0.020). DR5 was highly expressed in most cancer cells and some gastric cancer cells were confirmed with high DR5 expression in the nucleus. We considered that the malignant phenotype of DR5 could be mediated by its special localization in gastric cancer cells, which had covered up its apoptosis‐prompting function as localized in the cell membrane. Knockdown of DR5 could inhibit gastric cancer cells' growth and migration, but not of DR4. DR5, as a surface protein located at the cytomembrane of cancer cells, can receive and mediate TRAIL signals, inducing cancer apoptosis *in vivo*. This is evident from Fig. 3A,C, which showed that patients with negative DR4 expression had a worse OS outcome. To distinguish the role of DR5 from DR4, we focused on studying its function *in vitro* in the absence of TRAIL. We found that interfering with DR4 expression had no effect on gastric cancer cells *in vitro*, whereas interfering with DR5 expression did have an impact (Fig. 4). This suggests that DR5 may have a nonclassical function, independent of TRAIL, that promotes gastric cancer. This finding is consistent with the results shown in Fig. 4B,D,E. According to the result of Fig. 5, the xenograft mouse model *in vivo* further showed that mice injected with DR5‐knockdown cells had lower fluorescence intensity and pathological counting in metastatic foci mainly located in the lung, which confirmed that knockdown of DR5 could inhibit gastric cancer cells' metastasis and growth. Although the statistical differences in metastasis foci number were not obtainable (*P* = 0.403) due to the limitation of groups, a decreasing trend was evident. This might indicate that the role of DR5 in promoting gastric cancer metastasis is limited, and thus, the effect of solely intervening in DR5 may not be significant in reducing the individual metastatic rate. However, we confirmed that the nuclear localization of DR5 could play a continuous and important role in promoting the further proliferation of gastric cancer cells after entering metastatic sites, and it could weaken DR5 function in mediating tumor cell death induced by TRAIL. In general, gastric cancer cells with a high expression of DR5 were more aggressive and malignant *in vitro* and *in vivo*. The conclusion was consistent with other cancers, such as PDAC [15] and breast cancer [16].

DR4 and DR5 were found to be highly expressed with prognostic value in many cancers. In the past two decades, much research was mainly focused on the apoptosis‐promoting mechanism of DR4 and DR5 in cancer cells, which usually was not thought conducive to the survival of tumor cells. In the TRAIL‐induced apoptosis pathways, DR4 and DR5 binding with TRAIL in the extracellular region will recruit Fas‐associated protein with death domain (FADD) and procaspase‐8 to form a death‐inducing signaling complex (DISC), whose caspase‐8 cleavage then activates caspase‐3 and ultimately leads to apoptosis and death through caspase cascade [3]. In addition, caspase‐8 can also strengthen apoptosis through the pathway of Bid/Bcl‐2/Cytochrome‐C in a mitochondria‐dependent manner [4]. The mechanism had been further supported by the various cancers' clinical cohorts. For instance, in colon cancer, DR4 was confirmed significantly correlated with disease‐free survival [18]. In glioblastoma, DR5 had higher expression than DR4, and both were beneficial for survival as independent prognostic factors [19]. Nevertheless, in some cancers, the negative role of DR4 or DR5 was also reported. It was reported that DR4 was correlated with poor OS and progression‐free survival in ovarian cancer [20]. Despite the apoptosis‐prompting function of DR5, the DR5 level was too low to mediate an extensive effect of TRAIL‐induced killing [21, 22]. It was first reported that positive expression of DR5 in NSCLC was correlated with an increased death risk [10]. In head and neck squamous cell carcinoma, it was supposed that DR5 might be involved in metastasis [23]. In our previous study, however, we found that DR4 and DR5 had different roles in gastric cancer and that high DR4 expression was the main factor of enhancing apoptosis‐induced tumor‐killing effects in cells, but DR5 expression was not correlated with the apoptotic effect [7, 8]. Therefore, there should be some under‐discovered functions and roles of death receptors in cancer cells, and their biological significance might be completely different among the kinds of cancers.

Some researchers reported the nonclassical function of DR5 in the nucleus of some cancer cells. One study [14] reported that TRAIL could interact with DR5 and activate the NF‐κB pathway in B16F10 (mouse melanoma cells), and then induced MMP‐9 that can promote tumor proliferation and lung metastasis *in vivo*. Afterward, one study [15] found that TRAIL/DR5 could interact with Rac1/PI3K/AKT and then induce proliferation and metastasis in KRAS‐mutated cells (NSCLC and PDAC tumor cells). It was further confirmed that DR5 could promote AKT phosphorylation and glucose uptake in nontumor cells [24]. To some degree, this could explain the negative impact of DR5 on survival, but the TRAIL/DR5/NF‐κB or TRAIL/DR5/Rac1/PI3K/AKT pathway was still not enough to elucidate the malignant phenotype of tumor cells with high DR5 expression. Nevertheless, one study reported that importin β1 was responsible for the nuclear localization of DR5 containing nuclear localization signals (NLS) in Hela and HepG2, which limited DR5‐induced apoptosis and cell death [12]. They inferred that tumor cells without DR5 in the nucleus were sensitive to TRAIL and those with DR5 in the nucleus were resistant. In addition, inhibition of importin β1 could enhance anti‐DR5 treatment in TRAIL‐resistant cancer cells [25]. The mechanism of DR5 translocation into the cell nucleus provided a whole new understanding of the role of DR5, which then was confirmed [13], who revealed that DR5 in the nucleus of PDAC cells could inhibit let‐7 maturation and then promote cell proliferation through strongly inducing expression of HMGA2 and Lin28B, which is targeted by let‐7. One study [16] reported that DR5 could upregulate CXCR4 and then enhance SDF‐1‐directed migration in breast cancer cells. They supposed the mechanism was based on the mechanism of DR5/let‐7 in the cell nucleus. We further analyzed the genes mentioned above (such as MMP9, HMGA2, and KPNB1) in the gastric cancer cohort (TCGA‐STAD), and found that DR5 was positively correlated with them and higher than DR4.

Unlike other types of cancer cells, gastric cancer cells were resistant to TRAIL‐induced apoptosis. It was reported that the high level of active Akt and FLICE inhibitory protein (FLIP) in gastric cancer cells rendered resistance [26]. CbI regulating DR5 distribution in lipid rafts could also reduce sensitivity to TRAIL‐induced apoptosis [27]. However, they could not explain the results of DR5‐related malignant phenotype or poor prognosis. This was the first study to report that DR5 in the nucleus presented the aggressive behaviors and a worse prognosis in gastric cancer. Concerning the research of DR5 in gastric cancer, many of them mainly focused on the compounds' capability to sensitize TRAIL‐induced apoptosis by overexpression of DR5 or regulation of the TRAIL/DR5 pathway [28]. However, dual locations and effects of DR5 might be ignored. Admittedly, the sensitivity of the cells to the drug was enhanced due to TRAIL/DR5‐related apoptosis, but perhaps DR5 in cells' nuclei inducing aggressive behaviors was more serious for increasing the invasion and metastasis.

Nevertheless, several questions remain open. First, the detailed mechanism needs further studies. It was unexplained how DR5 was regulated and translocated and how it could drive cell invasion and migration in the nuclear localization of gastric cancer cells. Second, DR5 in the nucleus might also be involved in immune resistance, m6A regulation, TMB, and MSI, but the detailed mechanisms and significance are still unclear. Third, although DR5 presented aggressive behavior and was correlated with poor survival outcomes, recombinant human TRAIL (rhTRAIL) could still interact with DR5 and induce related apoptosis [29]. The different sensitivity to rhTRAIL among cancers may depend on their unique biological characteristics. Therefore, due to the dual effects of DR5 on apoptosis and proliferation, it should be further verified whether DR5 is appropriate as an intervention target or an additional regulated objective for gastric cancer treatment.

## Materials and methods

### Bioinformatic analysis

The data on transcriptomic expression in cell lines were obtained and analyzed from The Cancer Genome Atlas (TCGA, https://portal.gdc.cancer.gov/) and Cancer Cell Line Encyclopedia (CCLE, https://sites.broadinstitute.org/ccle/). The data of transcriptomic expression profiles in patient samples and clinical information were downloaded from the TCGA. Spearman's correlation in multiple‐gene correlation was used to describe correlation. ‘Adjusted *P* value < 0.05 and the absolute value of Log_2_ (Fold‐Change) > 1’ were defined as the threshold for the differential expression. For a better understanding of enriched gene functions, Gene Ontology (GO) and Kyoto Encyclopedia of Genes and Genomes (KEGG) enrichment analyses were performed. The mRNAsi score was calculated using the one‐class logistic regression (OCLR) algorithm [30]. The correlation analysis between gene expression and tumor mutation burden (TMB) and microsatellite instability (MSI) score were performed using Spearman's method. The data of gene expression in response to immune checkpoint inhibitor (ICI) were obtained from IMvigor210 [17]. The single‐cell RNA‐Seq (scRNA‐Seq) data were obtained from GSE163558 [31]. Through quality control and filtration, ultimately 37,274 cells with 26,089 genes were included, which were from the six different samples containing adjacent normal tissue (NT), primary tumor (PT), metastatic tumor samples in lymph node (LN), ovary, peritoneum, and liver. Through reduction (tSNE and UMAP) and definition of clusters, the subcluster consisting of epithelial cells in NT and tumor cells from other five sources were picked out for further analysis, amounting to 1865 cells. The bioinformatics analyses were based on r software (v. 4.2.0, R Foundation for Statistical Computing, Vienna, Austria) and the r packages ‘seurat,’ ‘harmony,’ ‘clustree,’ ‘clusterprofiler,’ ‘limma,’ ‘heatmap,’ ‘ggplot2,’ and ‘ggpubr’ were used for the above analyses.

### Patient characteristics

Two hundred and forty patients, 69 males and 71 females, with gastric cancer were included, which were diagnosed and surgically treated at Peking University Cancer Hospital between 1996 and 2008. The mean age was 59.22 years (range, 22–81 years). After the operation, the tumor specimens were routinely pathologically assessed. The recorded clinicopathological information included gender, age, tumor differentiation of tumor (well, moderate, poor), histologic type of tumor (adenocarcinoma or others), lymphovascular invasion (absent or present), depth of invasion (T1, T2, T3, and T4) by the 7th tumor‐node‐metastasis (TNM) classification recommended by the American Joint Committee on Cancer, lymph node metastasis (no or yes), distant metastasis by TNM classification (M0 or M1), and T and NM stage (I, II, III, IV). The human study was approved by the Ethics Committee of Peking University (2019kt111) and written informed consent in compliance with the Declaration of Helsinki from each patient was obtained.

### Ethics approval

The human study was approved by the Ethics Committee of Peking University (2019kt111) and written informed consent in compliance with the Declaration of Helsinki from each patient was obtained. The animal study was approved by the Ethics Committee of the Peking University Aerospace School of Clinical Medicine (nos.CR‐20200826‐NSFC‐03) in accordance with the Guide for the Care and Use of Laboratory Animals of the National Institutes of Health (NIH, Bethesda, MD, USA).

### Tissue specimens and immunohistochemistry staining

Tissue specimens from patients during surgery were obtained. Connective tissues and necrotic hemorrhage were removed. For immunohistochemistry (IHC), tissues were first processed to be formalin‐fixed paraffin‐embedding (FFPE) specimens. Then, FFPE sections with 5 μm thickness were in the tissue microassay (TMA), including 240 gastric cancer tissue samples. The slides were deparaffinized with xylene and rehydrated by graded alcohol. The slides in 3% H_2_O_2_ for 10 min were aimed at blocking endogenous peroxidase activity. Then the slides were processed in sequence through antigen retrieval in boiled alkaline Tris buffer, 5% milk blocking, incubation of primary antibody, incubation of secondary antibody, dehydration, and sealing. The primary antibody of DR4 was from Abcam (#ab8414, Cambridge Biomedical Campus, Cambridge, UK) and DR5 from Sigma‐Aldrich (St. Louis, MO, USA; #HPA023625), both at dilution of 1:1000. REALTM EnVision^TM^ (Dako, Carpinteria, CA, USA) was used as a secondary antibody and DAB chromogen for color development. The expression of DR4 or DR5 was assessed independently by two experienced pathologists. The intensity of the staining was categorized as negative (−) or positive (+). High consistency and consensus were reached between two pathologists (discrepancy rate < 5%).

### Cell culture and reagents

Gastric cancer cell lines (SGC7901, BGC823, MKN74, and N87) were used in this study and purchased from the Cell Research Institute (Shanghai, China) and the ATCC. High‐glucose Dulbecco's Modified Eagle Media (DMEM, Gibco, New York, NY, USA) and Roswell Park Memorial Institute medium 1640 (RPMI‐1640, Gibco) were used as the medium for cell culture. The cells were cultured in DMEM or RPMI‐1640 with 10% (v/v) fetal bovine serum (FBS) (Gibco) and incubated at 37 °C in a humidified 5% CO_2_ atmosphere.

### Cell viability assay

Cells were inoculated in a 96‐well plate with a density of 3000 cells per well. Cell viability was measured using CCK‐8 (DOJINDO, Kumamoto, Japan) by incubating cells for 2 h at 37 °C on Days 0, 1, 2, 3, and 4. A microplate reader was used to measure the absorbance of wells with CCK‐8 solution at 450 nm.

### Western blotting (WB) assay

Cells were partitioned into nuclear and cytoplasmic fractions with a Nuclear Protein Extraction Kit (Solarbio Life Science, Beijing, China) according to the manufacturer's instructions. The samples were separated through 12% SDS polyacrylamide gel electrophoresis (SDS/PAGE) and transferred onto a nitrocellulose membrane. Then the membrane was blocked for 1 h at room temperature using a buffer containing 5% nonfat milk and incubated with the following primary antibodies overnight at 4 °C: DR5 (#ab8416, Abcam), Lamin B1 (#12586, Cell Signaling Technologies, Danvers, MA, USA), and β‐actin (sc‐69879, Santa Cruz, Dallas, TX, USA). After being washed using Tris‐buffered saline tween‐20 (TBST), the membrane was incubated with the horseradish peroxidase (HRP)‐conjugated secondary antibodies. Signals were detected using chemiluminescent agents (#7003, Cell Signaling Technologies). The expression of proteins was quantified based on the gray‐scale value using imagej (NIH).

### RNA interference

The si‐Control (sc‐37007) and siRNA of DR4 (sc‐35218) or DR5 (sc‐40237) were purchased from Santa Cruz. Multiple siRNAs were pooled to interfere with the cancer cells according to the manual. The negative control (NC) and shRNA of DR5 (KD1: 5′‐CACCCTGGAGTGACATCGAAT‐3′; KD2: 5′‐CAGGGACACCTTGTACACGAT‐3′; KD3: 5′‐GTCCCTGAGCAGGAAATGGAA‐3′) were synthesized by Genechem (Shanghai, China). Concerning siRNA transfection, cells were inoculated into a 6‐well plate and then transfected with NC or siRNA using lipofectamine 2000 (Invitrogen, Waltham, MA, USA) in FBS‐free DMEM for 6 h, reaching 70–80% confluence. Concerning shRNA, lentivirus was used to prepare plasmid vectors that infected gastric cancer cells.

### Transwell assays of migration

Cells were plated into the top chamber in the migration assay. The bottom chamber was filled with the DMEM medium. After 48 h incubation, the cells were stained with 0.1% crystal violet for 20 min. Then the cells on the underside of the membrane were counted by microscope for assays of cell migration.

### *In vivo* xenograft mouse model

This study was strictly performed in accordance with the Guide for the Care and Use of Laboratory Animals of the NIH. The Ethics Committee of the Peking University Aerospace School of Clinical Medicine approved animal protocols in this study (nos.CR‐20200826‐NSFC‐03). For assessing the effect of DR5 on metastasis, the BGC823 cells transfected with NC or shRNA‐DR5 were injected intravenously into the tail vein of female BALB/C‐nude mice at 5 weeks (2 × 10^6^ cells in 100 μL phosphate‐buffered saline [PBS] buffer per mouse). All the mice were anesthetized in diethyl ether for *in vivo* fluorescence imaging, and the total radiant efficiency was recorded. The mice were euthanized through cervical dislocation.

### Statistical analysis

All the data are presented as the mean ± standard deviation (SD). A standard chi‐squared test was used to assess the relationship between DR4/5 expression and the clinicopathological features of patients with gastric cancer. Kaplan–Meier curves were drawn to present the survival outcomes, and the log‐rank test was used to compare survival differences. Overall survival was defined as the time from the date of surgery to the date of the last follow‐up or death. The influence of each variable on survival was assessed using univariate and multivariate analysis of the Cox proportional hazards regression model. The software graphpad prism (GraphPad Prism 8, Palo Alto, CA, USA) was used for experimental results analysis and outcomes presentation. Student's *t*‐test was used to compare two groups. All tests were two‐tailed. *P* < 0.05 was significantly different.

## Conflict of interest

The authors declare no conflicts of interest. The funders had no role in the design of the study; in the collection, analyses, or interpretation of data; in the writing of the article; or in the decision to publish the results.

### Peer review

The peer review history for this article is available at https://www.webofscience.com/api/gateway/wos/peer‐review/10.1002/2211‐5463.13725.

## Author contributions

LL, JC, XX, and LH contributed to the conception and design. XX, XJ, and JJ provided administrative support. XJ and XX were involved in the provision of study materials or patients. LL, JC, HD, and LH contributed to the collection and assembly of data. LL, JC, and LH were involved in the data analysis and interpretation. JC, XX, and LH were involved in the article writing. JC and LL contributed equally as the co‐first author. All authors have read and agreed to the published version of the article.

## Supporting information


**Fig. S1.** Comparison of DR4 expression between groups of nonresponse and response in the IMvigor210 cohort. Mann–Whitney *U*‐test was used.

**Fig. S2.** Original images for blots and gels. (A) The original image of DR5 band in Fig. 4C; (B) The original image of Lamin B1 band in Fig. 4C.

## Data Availability

No raw data were created for this paper. The bioinformatic data included and analyzed in this study are available in the TCGA database (https://portal.gdc.cancer.gov/) and CCLE database (https://sites.broadinstitute.org/ccle/). The other data were obtained from GSE163558 and IMvigor210.
