# Supplementary material for: Death receptor 5 promotes tumor progression in gastric cancer
Source: FEBS Open Bio. 2023 Nov 14;13(12):2375–88. doi: 10.1002/2211-5463.13725 (PMC10699099; doi:10.1002/2211-5463.13725)

Figure S1. The comparison of DR4 expression between groups of non-response and response in the IMvigor210 cohort. Mann-Whitney U test was used.

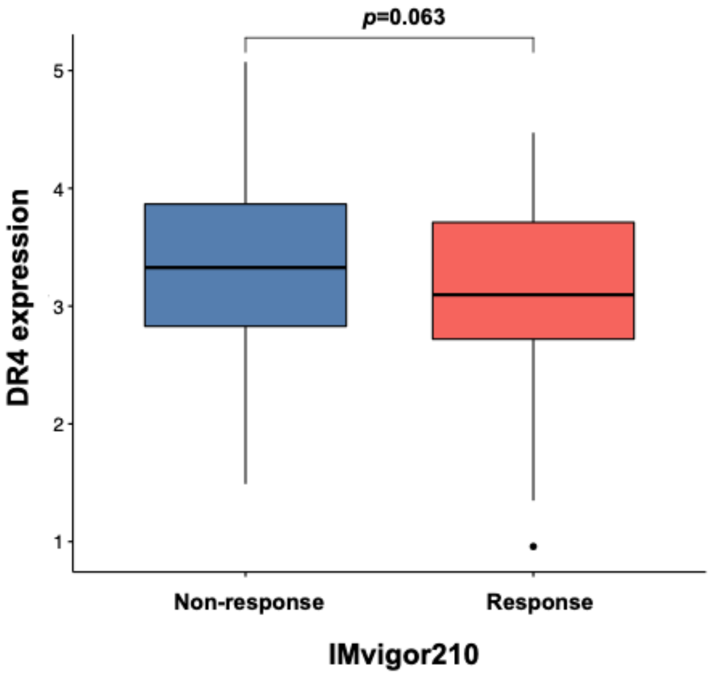

Supplement: Supplementary file 1 — Fig. S1. Comparison of DR4 expression between groups of nonresponse and response in the IMvigor210 cohort. Mann–Whitney U‐test was used. [file FEB4-13-2375-s001.pdf]
