# Supplementary material for: Death receptor 5 promotes tumor progression in gastric cancer
Source: FEBS Open Bio. 2023 Nov 14;13(12):2375–88. doi: 10.1002/2211-5463.13725 (PMC10699099; doi:10.1002/2211-5463.13725)

Figure S2. The original images for blots and gels. (A) The original image of DR5 band in Figure 4C; (B) The original image of Lamin B1 band in Figure 4C.

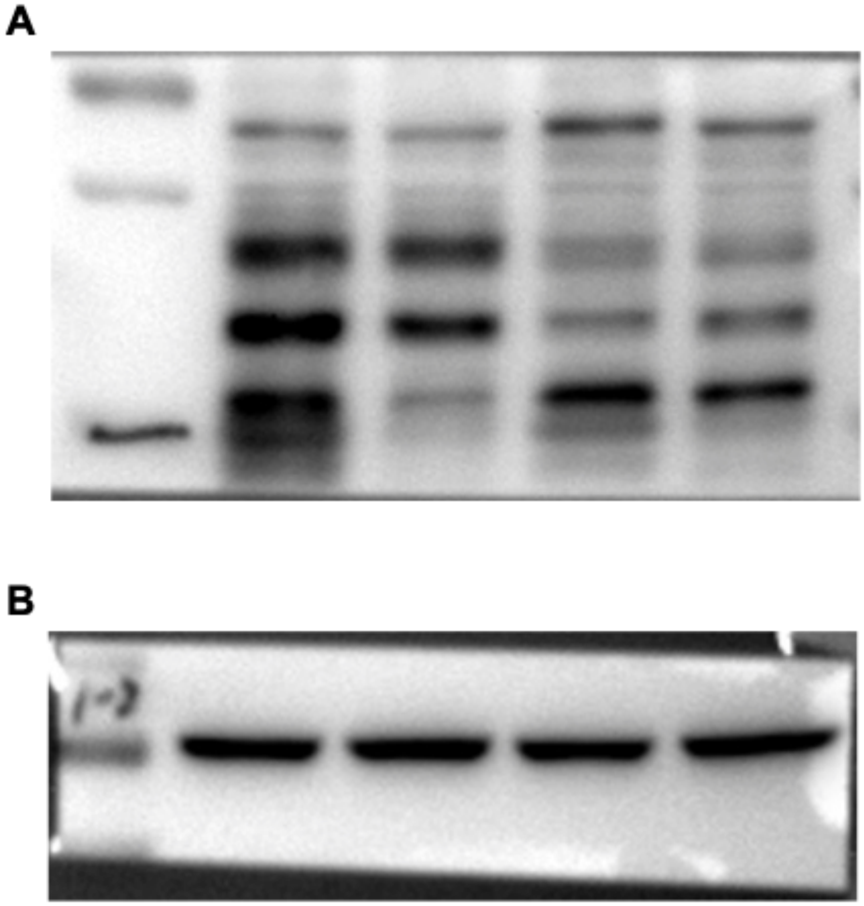

Supplement: Supplementary file 2 — Fig. S2. Original images for blots and gels. (A) The original image of DR5 band in Fig. 4C; (B) The original image of Lamin B1 band in Fig. 4C. [file FEB4-13-2375-s002.pdf]
